# Rapid Determination of Thiabendazole Pesticides in Rape by Surface Enhanced Raman Spectroscopy

**DOI:** 10.3390/s18041082

**Published:** 2018-04-04

**Authors:** Lei Lin, Tao Dong, Pengcheng Nie, Fangfang Qu, Yong He, Bingquan Chu, Shupei Xiao

**Affiliations:** 1 College of Biosystems Engineering and Food Science, Zhejiang University, Hangzhou 310058, China; linlei2016@zju.edu.cn (L.L.); 21613052@zju.edu.cn (T.D.); npc2012@zju.edu.cn (P.N.); ffqu@zju.edu.cn (F.Q.); chubingquan10@163.com (B.C.); 180312@zju.edu.cn (S.X.); 2 Key Laboratory of Sensors Sensing, Ministry of Agriculture, Hangzhou 310058, China; 3 State Key Laboratory of Modern Optical Instrumentation, Zhejiang University, Hangzhou 310058, China

**Keywords:** Surface Enhanced Raman Spectroscopy (SERS), rape, thiabendazole pesticides, PLS, rapid detection

## Abstract

Thiabendazole is widely used in sclerotium blight, downy mildew and black rot prevention and treatment in rape. Accurate monitoring of thiabendazole pesticides in plants will prevent potential adverse effects to the Environment and human health. Surface Enhanced Raman Spectroscopy (SERS) is a highly sensitive fingerprint with the advantages of simple operation, convenient portability and high detection efficiency. In this paper, a rapid determination method of thiabendazole pesticides in rape was conducted combining SERS with chemometric methods. The original SERS were pretreated and the partial least squares (PLS) was applied to establish the prediction model between SERS and thiabendazole pesticides in rape. As a result, the SERS enhancing effect based on silver Nano-substrate was better than that of gold Nano-substrate, where the detection limit of thiabendazole pesticides in rape could reach 0.1 mg/L. Moreover, 782, 1007 and 1576 cm^−1^ could be determined as thiabendazole pesticides Raman characteristic peaks in rape. The prediction effect of thiabendazole pesticides in rape was the best ($R_{p}^{2}$ = 0.94, *RMSEP* = 3.17 mg/L) after the original spectra preprocessed with 1st-Derivative, and the linear relevance between thiabendazole pesticides concentration and Raman peak intensity at 782 cm^−1^ was the highest (*R^2^* = 0.91). Furthermore, five rape samples with unknown thiabendazole pesticides concentration were used to verify the accuracy and reliability of this method. It was showed that prediction relative standard deviation was 0.70–9.85%, recovery rate was 94.71–118.92% and *t* value was −1.489. In conclusion, the thiabendazole pesticides in rape could be rapidly and accurately detected by SERS, which was beneficial to provide a rapid, accurate and reliable scheme for the detection of pesticides residues in agriculture products.

## 1. Introduction

Thiabendazole, a benzimidazole derivative which belongs to the absorption of a broad-spectrum fungicide, has been widely used in rape sclerotium blight, downy mildew and black rot disease prevention. The long-term exposure or misuse of thiabendazole pesticides could lead to a variety diseases such as cancer, blood diseases and immune system disorders, which seriously affects the physical and mental health [1]. In accordance with the provisions of the maximal residue of pesticides in food in China (GB 2763-2014), the thiabendazole pesticides in rape cannot exceed 1 mg/kg. Traditional methods for determining thiabendazole pesticides are mainly include high-performance liquid chromatography (HPLC) [2], fluorescence quantification [3], ion exchange chromatography [4] and capillary electrophoresis [5]. Šmídová et al. [6] successfully applied immunochromatographic technique to the detection of the pesticides thiabendazole and methiocarb in fruit juices. Chen et al. [7] achieved the determination of residues of carbendazim and thiabendazole pesticides in fruits by dispersive solid-phase extraction and HPLC. The results showed that the regression analysis for the pesticides were linear at concentrations ranging from 0.01 mg/kg to 3.0 mg/kg, and the correlation coefficient was higher than 0.999. Although the sensitivities of these methods are high, the cumbersome pre-test, time-consuming detection, inconvenient instrument, expensive reagents and other shortcomings limit their developments [8].

SERS is a technique that enhances the intensity of Raman signals with increasing orders of magnitude. Its compound molecule signal can be enhanced in geometric multiple when it adsorbs on some nanoscale rough metals surface (such as gold, silver and copper) [9]. Besides, SERS has the advantages of simple pretreatment method, convenient equipment and fast detection speed, which is suitable for rapid screening of molecule substances [10]. As the aspects of the thiabendazole pesticides residues detection in agriculture, many domestic and foreign scholars have carried out related researches. As for the Raman peaks of thiabendazole pesticides, Lin et al. [11] applied the SERS with silver nanoparticle technique to analyze the Raman peaks of thiabendazole pesticides. It was indicated that the characteristic Raman peaks at 782, 1012, 1284, 1450 and 1592 cm^−1^ belonged to the thiabendazole pesticides. The detection line of thiabendazole residues in apples has been studied. Luo et al. [12] achieved the rapid detection of phosmet and thiabendazole residues in apples using SERS coupled with gold nanoparticles. The results suggested that the minimum detectable concentration was 0.5 mg/g for phosmet and 0.1 mg/g for thiabendazole in apples. In addition, rapid detection of thiabendazole has showed a great significance in agriculture production. Feng et al. [13] combined molecularly imprinted polymers with SERS to determine trace amount of thiabendazole in orange juice. The whole detection was only took 23 min with limit of detection of 4 ppm for thiabendazole. He et al. [14] quantitatively detected the thiabendazole on apples using a surface swab capture method followed by SERS, which indicated that the swab-SERS method was simple, sensitive, rapid (10 min), and quantitatively enough detect other pesticides on raw agricultural produce like pears, carrots, and melons etc. Beside these, the miniaturization and portability of Raman detection are becoming more and more important. Müller et al. [15] detected the thiabendazole in citrus fruits and bananas using SERS with a compact, portable, mini-Raman spectrometer, which estimated a total amount of thiabendazole as 78 mg/kg in citrus fruits. Although the above researches achieved the purpose of enhancing the signal of thiabendazole pesticide molecules by SERS in agricultural products, the effect of matrix substances could not be fully removed and the detection limit of the method should be further improved as well as the accuracy and stability. Moreover, there are little reports about the detection of thiabendazole pesticides residues in rape by the combination of SERS and chemometric method.

Therefore, the main objective of this paper is to detect thiabendazole pesticides in rape rapidly and accurately using SERS combined with chemometric methods. Moreover, the fluorescent effects of chlorophyll, fat, carbohydrates and other substances in rape were removed using ferroferric oxide (Fe_3_O_4_) nanoparticles [16], which was of great significance to provide a rapid-detection method of pesticides residues with the advantages of low cost, easy operation and high accuracy.

## 2. Materials and Methods

### 2.1. Experimental Instruments and Reagents

For this study, the experimental instruments mainly included: (1) RmTracer-200-HS portable Raman spectrometer combined with a 785 nm excitation wavelength diode-stabilized stimulator (Opto Trace Technologies, Inc., San Francisco Bay Area, CA, USA); (2) JW-1024 low-speed centrifuge (Anhui Jia Instrument and Equipment Co., Ltd., HeFei, Anhui, China); (3) Vortex-Genie 2/2T vortex mixer (Shanghai Ling early Environmental Protection Instrument Co., Ltd., ShangHai, China); (4) Agilent 1290 Ultra Performance Liquid Chromatography Combined Photodiode Array Detector (Agilent Technologies, Santa Clara, CA, USA); (5) The column, Agilent ZORBAX SB-C18, 150 mm × 2.1 mm, 3.5 μm (Agilent Technologies, Santa Clara, CA, USA); (6) The FEI Tecnai G2 F20 S-TWIN transmission electron microscope (TEM, USA FEI Corporation, Hillsboro, OR, USA); (7) ZNCL intelligent thermostat magnetic stirrer (Zhengzhou Ya-Rong Instrument Co., Ltd., Zhengzhou, China). 

Moreover, the experimental reagents included: (1) thiabendazole (99.7% purity, Sigma-Aldrich, Beijing century Aoke Biological Technology Co. Ltd. Beijing, China); (2) acetonitrile (chromatographically purity, Amethyst Chemicals. Beijing century Aoke Biological Technology Co. Ltd., Beijing, China); (3) Ferroferric oxide (100 nm particles, Hangzhou Wan King New Material Co., Ltd., Hangzhou, China); (4) Sodium chloride (Analytical Pure, National Standards Information Center, Beijing, China); (5) Silver nitrate, perchlorate, trisodium citrate, primary secondary amine (PSA) (ethylenediamine -*N*-propyls lane), anhydrous magnesium sulfate, C18 and graphitized carbon (Agela Technologies, Tianjin, China); (6) Organic filter (0.22 μm, Agilent Technologies, Inc., Santa Clara, CA, USA). 

### 2.2. Experimental Methods

#### 2.2.1. Sample Preparation

In this experiment, 101 pesticide-free rape plants leaf (high-oil 605) were selected as the experimental samples. The specific operations of the simulation of thiabendazole pesticides residues in rape were as follows. First, 101 different concentrations thiabendazole standard solutions (0–100 mg/L, 1 mg/L per gradient) were prepared and then sprayed on 101 pesticide-free rape plants leaf. The rapes were numbered from 1 to 100 in sequence, where 0 was set as a blank contrast. Second, the corresponding rape leaf samples were picked after 24 h natural simulation spraying. Third, the Raman detection samples were prepared: 10 g rape leaf (each sample), 10 mL acetonitrile, 5 g sodium chloride were mixed and stirred, then the sample supernatant was obtained after centrifugation. Fourth, 600 mg ferroferric oxide (Fe_3_O_4_) nanoparticles was added into the supernatant to remove the fluorescent effects of chlorophyll, fat, carbohydrates and other substances in rape. Finally, the supernatant was filtered by a 0.22 μm organic membrane. 

#### 2.2.2. Silver Nano-Substrate and Gold Nano-Substrate Preparation

The preparation of silver nanoparticle was established on the basis of the Lee–Meisel trisodium citrate heating reduction method [17]. The silver Nano-substrate preparation process was as follows. First, a silver nitrate solution (180 mg/L) was heated quickly for boiling at the high temperature on a constant temperature magnetic stirrer. Second, 1% trisodium citrate solution was dropwise added and stirred at 200 r/min. Third, it was stored in dark after the solution turned to gray-green in 25 min. Fourth, when the heating temperature was dropping, the silver nitrate solution was poured into a centrifuge tube and then a small amount of supernatant was remained after centrifugation. Finally, ultrapure water was mixed with ultrasonic vibration, and the gold colloid was stored in dark at 4 °C after repeating purification.

The trisodium citrate heating reduction method was slightly modified according to the literature for the preparation of gold nanoparticle [18]. The gold Nano-substrate preparation process was as follows. First, a chloroauric acid solution (50 mg/L) was heated for boiling at 120 °C on a constant temperature magnetic stirrer, and then 4 mL of trisodium citrate solution (5 mg/mL) was added. Second, the mixture was stirred at 100 r/min until the gold sol change into the color of the wine red. Third, when the solution cooled, the gold gel solution was poured into a centrifuge tube. Fourth, the supernatant (2 mL) was remained after centrifugation. Finally, 1 mL ultrapure water was added to the centrifuge tube and mixed with ultrasonic oscillation and the gold colloid was stored in dark at 4 °C after repeating purification.

#### 2.2.3. Thiabendazole Pesticides in Rape Detection

Thiabendazole pesticides in rape were detected by Ultra high performance liquid chromatography (UHPLC) [19]. The supernatant was prepared as Raman samples preparation in Section 2.2.1. The detection operation was as follows. First, 2 mL supernatant, 150 mg magnesium sulfate, 50 mg primary secondary amine (PSA), 10 mg graphitized carbon black and 50 mg C_18_ were added into the 15 mL centrifugal tube in sequence and then were centrifuged for 5 min at 4200 r/min. Second, 1 mL supernatant was put into a 10 mL glass and 1 mL ethyl acetate was added after the drying of nitrogen. Third, the supernatant was filtered by 0.22 μm organic membrane after 1 min vortex. 

### 2.3. Raman Spectrum Acquisition

Before RS data acquisition, the instrument was calibrated using a 785 nm excitation wavelength. The parameters were as follows: a power of 200 mw, a scanning range of 200 to 3300 cm^−1^, an optical resolution of 2 cm^−1^, an integration time of 10 s and an average spectral value of 3 times. The solid thiabendazole RS collection was that thiabendazole powder was in quartz plate with glass slides flattened and the spectra were acquired with matching microscope platform. The SERS collection method was that 500 μL silver colloid, 100 μL test solution and 500 μL sodium chloride were added in turn into a 2 mL quartz bottle and then it was placed at a liquid sample pool.

### 2.4. Data Analysis

The noise caused by equipment and the interference of the fluorescence background in Raman signal could affect the detection results [20]. Therefore, standard normal variation (SNV), normalization (Nor), multiplicative scatter correction (MSC) and 1st-Derivative (1st-Der) were used to pretreat the original spectra and then PLS was used to model and analyze the spectral data. Besides, the thiabendazole characteristic peaks were also calculated by Density Functional Theory (DFT) to validate the reliability of SERS. In this paper, all data analyses were based on MATLAB R2014a (The Math-Works, Natick, MA, USA), Gaussian.v09 (Gaussian, Inc., Wallingford, CT, USA, 2009), OMNIC v8.2 (Infrared spectrum processing software, Thermo Fisher Scientific, Madison, WI, USA), Origin v8.0 (Electronic Arts Inc., Hampton, MA, USA), SPSS V17.0 platform (Statistical analysis software, IBM Corporation, Armonk, NY, USA).

#### 2.4.1. Spectral Preprocessing Methods 

For this paper, four spectral preprocessing methods were applied to dealing with the original spectra. The principle of SNV [21] algorithm is that the absorbance values of each wavelength point satisfy a certain distribution in each spectra, and the spectral correction was performed according to this assumption. Normalization [22] is a non-dimensional treatment means that the absolute value of the physical system into some relative value relationship, which simplify the calculation. The basic idea of MSC [23] algorithm is to use an ideal spectra to represent all the samples, and the original spectra is corrected with the slope and intercept of the linear equation. 1st-Derivative [24] can eliminate interference from other backgrounds and distinguish the overlapping peaks, which improves spectral resolution, sensitivity and the signal to noise ratio of the spectra to a certain extent. 

#### 2.4.2. Modeling Method

Partial least squares (PLS) is a most widely-used regression modeling method in spectral data analysis for its flexibility and reliability in dealing with the redundant spectral data [25]. When PLS is applied to dealing with the spectral data, the spectral matrix is decomposed first and the main principal component variables are obtained, then the contribution rate of each principal component is calculated. The flexibility of PLS makes it possible to establish a regression model in the case where the number of samples is less than the number of variables. In this study, the PLS model was established with the spectral data as *X* and the measured moisture content of thiabendazole pesticides as *Y*, whose best principal factor was determined by root mean square error of cross validation (RMSECV)

#### 2.4.3. Model Evaluation Index

In this experiment, the modeling effect is evaluated by the coefficient of determination (*R*^2^), the root mean square error (*RMSE*), relative standard deviation (*RSD*) and recovery rate (*RT*). The coefficient of determination *R*^2^ reflects the level of intimacy between variables, root mean square error *RMSE* reflects the accuracy of the model, *RSD* reflects the degree of discretization between individuals in the reflection group and recovery rate reflects the degree of coincidence between the results of the reaction and the true value. The closer the *R*^2^ to 1 and recovery rate ranges 80–120%, the lower the *RMSE* and *RSD*, the better the performance of the prediction model. In this paper, $R_{c}^{2}$ and $R_{p}^{2}$ represent the coefficients of determination of calibration set and prediction set respectively, $RMSE_{c}$ and $RMSE_{p}$ represent the root mean square error of the calibration set and prediction set, respectively [26,27]. 

#### 2.4.4. Density Functional Theory

Density Functional Theory (DFT), as a tool for calculating molecular energy and analyzing properties, has been widely used in the field of physics and chemistry. It provides the computational strategies for obtaining information about the energetics, structure, and properties of atoms and molecules [28,29]. Based on the idea that the electron density is the fundamental quantity for describing atomic and molecular ground states, Parr et al. [30] gave sharp definitions for chemical concepts in various branches of chemistry. Besides, the performance of Becke three-parameter Lee–Yang–Parr functional (B3LYP) functional in combination with various basis sets has been extensively tested for molecular geometries, vibrational frequencies, ionization energies and electron affinities, dipole and quadrupole moments, atomic charges, infrared intensities and magnetic properties [31]. Among the various of functions and basis sets in DFT, the hybrid functional B3LYP with the 6-31G (d,p) basis set has been commonly used in the Raman spectroscopic calculation of biological molecules [32,33]. In this paper, B3LYP/6-31G (d,p) was used for the theoretical simulation and calculation of thiabendazole molecules.

## 3. Results and Discussion

### 3.1. Thiabendazole SERS

**Figure 1:**
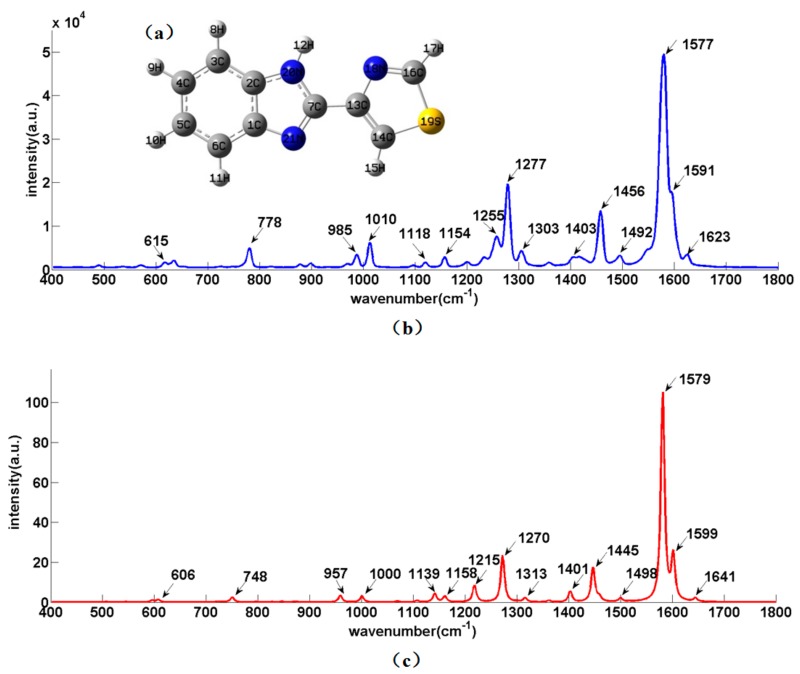
The molecular structure of thiabendazole and its positions of functional groups vibration. (**a**) The molecular structure of thiabendazole; (**b**) Raman Spectroscopy (RS) of thiabendazole solid; (**c**) the thiabendazole RS simulated by density functional theory (DFT).

Thiabendazole (molecular formula: C_10_H_7_N_3_S), consisted of benzimidazole and thiazole rings, is mainly composed of C–N, C=N, C=C, C–C, C–H, C–S and N–H groups. The molecular structure of thiabendazole is shown in Figure 1a. Figure 1b is the RS of solid thiabendazole and Figure 1c is the thiabendazole RS simulated by DFT, where the abscissa is the wavenumber and the ordinate is the SERS intensity.

According to Figure 1b,c, except for 748, 957, 1139, 1215 and 1641 cm^−1^, the solid thiabendazole Raman peaks were basically in line with those calculated by DFT, which indicated that the position of Raman peaks detected by SRES were feasible and reliable. Table 1 presents the proposed assignments of Raman peaks of thiabendazole. Among the thiabendazole solid Raman peaks, 615 cm^−1^ was the synergistic effects of C–C–C and S–C–N inner surface bending and deformable vibration. 778 cm^−1^ belonged to the surface bending vibration of C–H group. 985 cm^−1^ was the C–S stretching vibration. 1010, 1118, 1154 and 1303 cm^−1^ belonged to the bending vibration of C–H group surface. 1255 cm^−1^ was the ring vibration and 1277 cm^−1^ was the synergistic effects of ring vibration and C–H group surface vibration. 1403 cm^−1^ belonged to the stretching vibration of C=C bands. 1436, 1577, 1591 and 1623 cm^−1^ were the C=N stretching vibration and 1492 cm^−1^ was the synergistic effects of C=C stretching vibration and N–H inner surface bending vibration. It was concluded that these peaks could be used as thiabendazole solid Raman peaks, where the solid thiabendazole Raman peaks vibrated strongly at 778, 1010, 1277, 1456 and 1577 cm^−1^. The similar results could be found on the Lin’s research [11].

### 3.2. The Comparation of Silver Nano-Substrate and Gold Nano-Substrate

**Figure 2:**
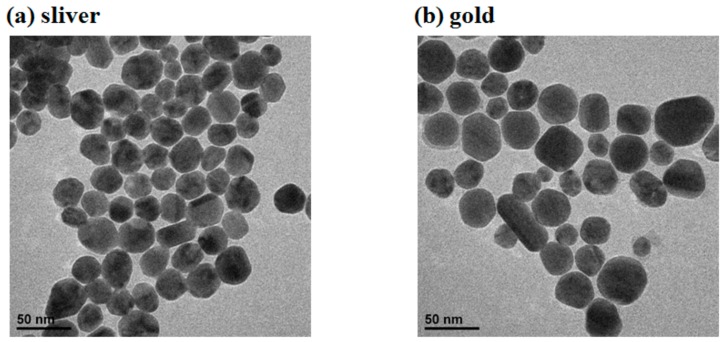
The structure and diameter of silver and gold nanoparticles: (**a**) Silver nanoparticle; (**b**) gold nanoparticle.

To investigate and find the optimal Nano enhancer, the enhancement effects of silver Nano-substrate and gold s Nano-substrate were compared and analyzed. The structure and diameter of silver nanoparticles and gold nanoparticles were characterized by Transmission Electron Microscopy (TEM) in Figure 2 respectively. 

**Figure 3:**
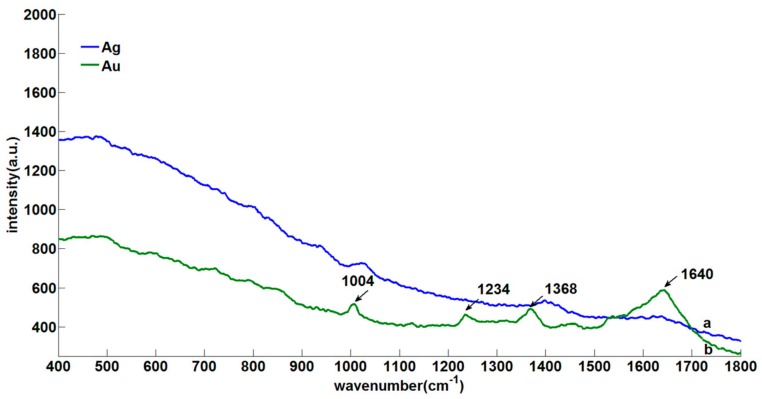
The RS of silver and gold substrate: (**a**) Silver Nano-substrate; (**b**) gold Nano-substrate.

Furthermore, the RS of silver substrate and gold substrate were analyzed to investigate whether the Nano enhancers had obvious Raman characteristic peaks. As can be seen from Figure 3, the diameter of silver nanoparticle was about 40 nm and that of gold nanoparticle was 60 nm, and the size of the two nanoparticles was uniform. Besides, there were almost no obvious Raman characteristic peaks in sliver Nano-substrate. Although there were few Raman characteristic peaks (1004, 1234, 1368 and 1640 cm^−1^) in gold Nano-substrate, the intensity strength was weak, which indicated that the two Nano enhancers could be used as the enhancement substrates in SERS.

### 3.3. The Enhancement of Silver Nano-Substrate and Gold Nano-Substrate

**Figure 4:**
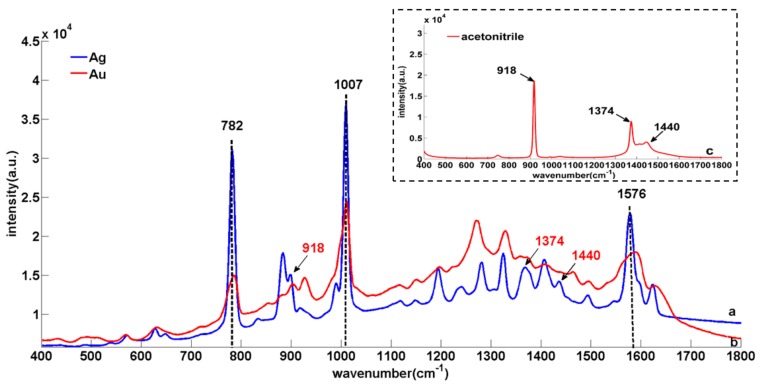
The Surface Enhanced Raman Spectroscopy (SERS) of the thiabendazole standard solution (100 mg/L) with silver and gold nanoparticle: (**a**) Silver; (**b**) gold; (**c**) acetonitrile.

The SERS of the thiabendazole standard solution (100 mg/L) with silver (Figure 4a) and gold Nano-substrate (Figure 4b) were compared and analyzed to determine which one performed better as Nano enhancer.

Given that the strong fluorescence interference produced by chlorophyll, protein, cellulose and other fluorescent substances in rape, the Fe_3_O_4_ nanoparticles (100 nm) with the advantage of low cost was added into the thiabendazole standard solution before the detection [16]. According to Figure 4, the enhancement effect of silver Nano-substrate on thiabendazole molecule was significantly higher at 782, 1007 and 1576 cm^−1^ than that of the gold Nano-substrate, which indicated that the enhanced performance of silver Nano-substrate thiabendazole molecules was better than that of gold Nano-substrate. On the one hand, the reason might be that the ultraviolet characteristic absorption peaks of thiabendazole and silver nanoparticles were more similar. Thus, the surface plasmon resonance of silver Nano-substrate can be stimulated more easily, resulting in the higher detection sensitivity [11] On the other hand, the mixture of Ag NPs with NaCl solution tends to form AgCl precipitates which affect the SERS drastically [34]. Beyond that, other Raman characteristic peaks are shown in Table 2. Except for those Raman peaks (918, 1374 and 1440 cm^−1^) which belonged to acetonitrile (Figure 4c), the thiabendazole solution characteristic peaks positions of SERS combined silver Nano-substrate and gold Nano-substrate were basically coincident with that of solid thiabendazole RS. Therefore, the silver Nano-substrate was selected to enhance the substrate in the following study.

### 3.4. Determination of Detection Line

**Figure 5:**
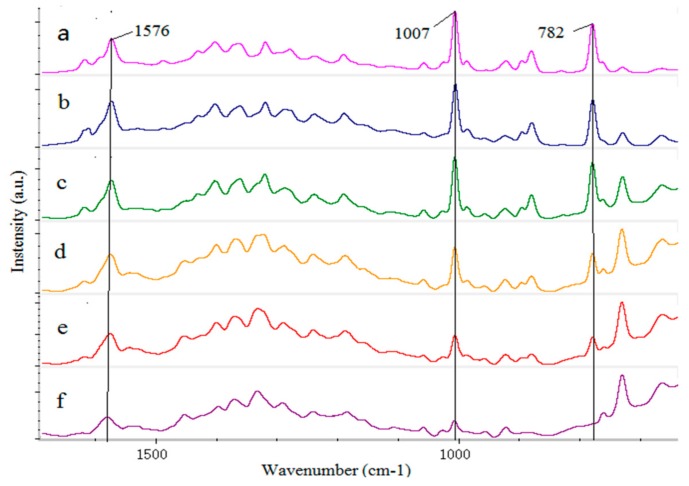
The SERS of thiabendazole pesticide solution in rape: (**a**) 10 mg/L; (**b**) 5 mg/L; (**c**) 1 mg/L; (**d**) 0.5 mg/L; (**e**) 0.1 mg/L; (**f**) 0.05 mg/L.

In order to further determine the detection line of thiabendazole pesticides in rape, six different concentrations thiabendazole pesticides (10, 5, 1, 0.5, 0.1, 0.05 mg/L) were prepared and the corresponding SERS are shown in Figure 5.

According to Figure 5, the SERS absorbance intensity decreased gradually with the decrease of thiabendazole pesticides concentration at the characteristic peaks of 782, 1007 and 1576 cm^−1^. When the concentration was 0.05 mg/L, the characteristic peaks signal was weak and only 1007 and 1576 cm^−1^ could be identified. Meanwhile, when thiabendazole pesticide was at 0.1 mg/L, the three characteristic peak signals were clearly visible. Thus, it was concluded that the detection limit of thiabendazole pesticides in rape was 0.1 mg/L using SERS and the detection limit was lower than the maximal pesticide residue of food (1 mg/L) in China, which indicated that the stoichiometric method could be used to quantitatively analyze the thiabendazole pesticide in rape.

### 3.5. The Analysis of SERS of Thiabendazole Pesticide in Rape

**Figure 6:**
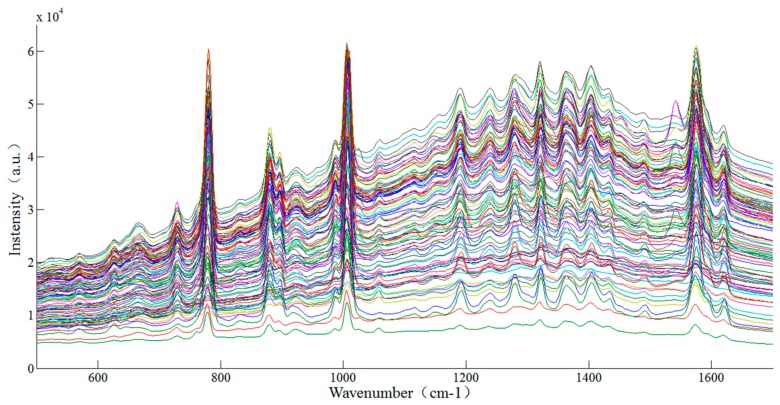
SERS spectra of different concentrations of thiabendazole pesticides in rape.

In this paper, four pretreatment methods were used to preprocess the original spectra and PLS was used for establishing the prediction model of thiabendazole pesticides in rape. The SERS of 101 samples are shown in Figure 6 and the modeling performance of different preprocessing methods are presented in Table 2. The sample set portioning based on joint x-y distance (SPXY) method [35] was used to divide the samples into two groups, among which 70 samples were calibrated and 31 samples were validated.

**Figure 7:**
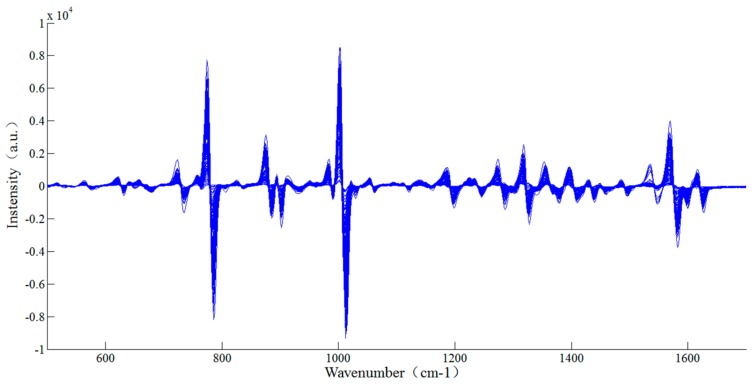
The pretreated spectra after the 1st-Der.

**Figure 8:**
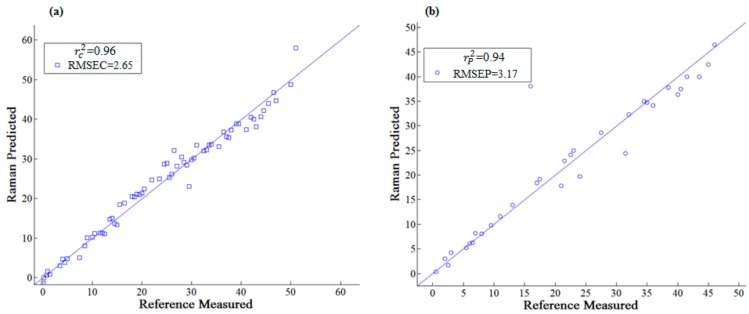
Scatter diagram of calibration set and prediction set by 1st-Der: (**a**) Calibration set; (**b**) prediction set.

It could be known from Table 2 that the prediction accuracy of thiabendazole pesticides in rape was the best ($R_{c}^{2}=0.96,\;RMSE_{c}=2.65\;\mathrm{mg}/L;R_{p}^{2}=0.94,\;RMSE_{p}=3.17\;\mathrm{mg}/L$) when the SERS were processed with 1st-Der (Figure 7) and the corresponding principal components was 5. The reason might be that the 1st-Der distinguished the overlapping peaks and eliminate interference from other backgrounds, which improved the spectral resolution and separated the main characteristic peaks (782, 1007 and 1576 cm^−1^) for quantitative analysis [24]. The scatter plot between the predicted values and the measured values of the correction set and the prediction set sample after the 1st-Der are shown in Figure 8. 

**Figure 9:**
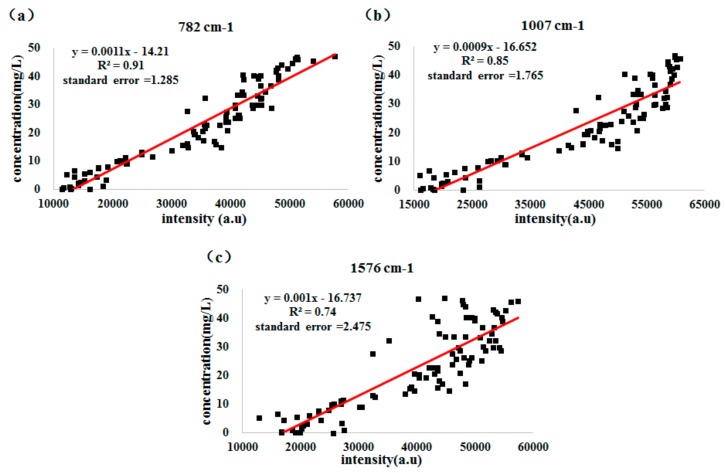
Regression equation of different characteristic band and SERS peak intensity. (**a**) Regression equation at 782 cm^−1^; (**b**) Regression equation at 1007 cm^−1^; (**c**) Regression equation at 1576 cm^−1^.

Figure 9 presents the regression equation between characteristic peaks (782, 1007 and 1576 cm^−1^) and SERS intensity, where the abscissa is the SERS intensity and the ordinate is the thiabendazole pesticides concentration in rape.

Among them, the coefficient of determination (*R*^2^ = 0.91) at 782 cm^−1^ was the highest and the regression equation was *y* = 0.0011*x* − 14.21. However, the coefficient of determination (*R*^2^) achieved 0.85 and 0.74 at 1007 and 1576 cm^−1^ respectively, which indicated that the thiabendazole pesticide concentration can be detected more accurately by SERS at 782 cm^−1^.

### 3.6. Model Accuracy Verification

#### 3.6.1. The Prediction Relative Standard Deviation and Recovery Rate

To verify the accuracy of the method, five rape samples with unknown thiabendazole pesticides concentration were pretreated using model and the true values of the five samples were detected by UHPLC. Table 3 presents the results between the real value and predicted value of thiabendazole pesticides in rape.

According to Table 3, the relative standard deviation between the true value and the predicted value ranged from 0.70% to 9.85% and the recovery was ranging from 94.71% to 118.92%. The results suggested that the predicted value was basically same as the measured value of UHPLC method, which indicated that the rapid detection of thiabendazole pesticide in rape by SERS was feasible.

#### 3.6.2. The *t* Test

Table 4 shows the results of the *t* test between the true values and the predicted values of the five unknown thiabendazole pesticide in rape. Among them, *t* value was −1.489 and its absolute value *df* = 3 was lower than *t*_0.05, 3_ = 3.182, which suggested that there was no significant difference between the true value and the predicted value. It was indicated that the rapid detection of thiabendazole pesticide residues in rape by SERS was reliable. 

## 4. Conclusions

In this paper, SERS technology was initially applied to detect thiabendazole pesticides in rape. The conclusions were as follows: (1) Thiabendazole pesticides in rape could be detected using SERS based on sliver Non-substrate and three Raman peaks (782, 1007 and 1576 cm^−1^) were selected as the characteristic peaks of thiabendazole pesticides in rape. The detection concentration could reach 0.1 mg/L. Among them, the thiabendazole pesticides prediction accuracy at 782 cm^−1^ was the highest (*R* ^2^= 0.91) and the regression equation was *y* = 0.0011*x* − 14. (2) The prediction results ($R_{p}^{2}$ = 0.94, *RMSE_P_* = 3.17 mg/L) was the best combined with 1st-Der preprocessing and PLS modeling. (3) Five rape samples with unknown thiabendazole pesticides concentration were pretreated to verify the accuracy of the method. It was indicated that the rapid detection of thiabendazole pesticides in rape by SERS was feasible and reliable. In conclusion, the rapid and accurate detection of thiabendazole pesticides in rape can be realized by SERS, which was conductive to provide a rapid and accurate scheme for the detection of pesticide residues.

## Figures and Tables

**Table 1:** The proposed assignment of Raman bands of thiabendazole.

Calculation (cm^−1^)	Solid (cm^−1^)	SERS-Ag (cm^−1^)	SERS-Au (cm^−1^)	Assignments
606 (w)	615 (w)	626 (w)	626 (w)	δ(C–C–C)ip δ(S–C–N)ip
748 (m)	778 (m)	782 (vs)	783 (m)	δ(C–H)oop
957 (w)	985 (w)	988 (m)	-	υ(C–S)
1000 (m)	1010 (m)	1007 (vs)	1007 (s)	δ(C–H)ip
1139 (w)	1118 (w)	1116 (w)	1116 (w)	δ(C–H)ip
1158 (m)	1154 (w)	1147 (m)	1147 (w)	δ(C–H)ip
1215 (w)	1255 (m)	1239 (w)	-	υ ring
1270 (s)	1277 (s)	1279 (m)	1270 (m)	υ ring + δ(C–H)ip
1313 (w)	1303 (w)	1322 (m)	1326 (m)	δ(C–H)ip
1401 (w)	1403 (w)	1404 (m)	1406 (m)	υ(C=C)
1445 (s)	1456 (s)	1433 (w)	1462 (m)	υ(C=N)
1498 (w)	1492 (w)	1492 (w)	1493 (w)	υ(C=C) + δ(N–H)ip
1579 (vs)	1577 (vs)	1576 (s)	1586 (s)	υ(C=N)
1599 (s)	1591 (s)	-	-	υ(C=N)
1641 (w)	1623 (w)	1621 (w)	1626 (w)	υ(C=N)

Note: vs = very strong; s = strong; m = medium; w = weak; υ = stretching; opp = outer surface bending; ip = Inner surface bending; δ = deformable vibration.

**Table 2:** The results of pre-processing method for calibration and prediction model.

Pre-Processing Method	Principal Components	Calibration		Prediction	
		$R_{c}^{2}$	RMSEC (mg/L)	$R_{p}^{2}$	RMSEP (mg/L)
Original	5	0.86	2.86	0.90	4.77
MSC	5	0.92	3.21	0.72	3.48
SNV	5	0.90	2.99	0.72	3.51
Normalization	5	0.86	4.32	0.79	3.44
1st-Der	5	0.96	2.65	0.94	3.17

**Table 3:** The results between the real values and predicted values of thiabendazole pesticides in rape.

Sample	Measured Value (mg/L)	Predicted Value (mg/L)	RSD (%)	Recovery (%)
1	1.432	1.703	9.85	118.92
2	6.234	5.904	0.70	94.71
3	10.231	9.987	3.22	97.62
4	20.431	21.233	1.15	103.93
5	33.156	34.187	7.96	103.11

**Table 4:** The *t*-test result between reference values and prediction values.

Paired *t* Test	Mean	Standard Deviation	*t* Value	*df*	Sig. (Two-Sided)
Measured value-predicted value	0.3726	0.5243	−1.489	3	0.978
